# Costs and cost-effectiveness of HIV early infant diagnosis in low- and middle-income countries: a scoping review

**DOI:** 10.1186/s40249-022-01006-7

**Published:** 2022-07-15

**Authors:** Kira Elsbernd, Karl M. F. Emmert-Fees, Amanda Erbe, Veronica Ottobrino, Arne Kroidl, Till Bärnighausen, Benjamin P. Geisler, Stefan Kohler

**Affiliations:** 1 Division of Infectious Diseases and Tropical Medicine, Faculty of Medicine, Ludwig Maximilians University, Munich, Germany; 2 Institute for Medical Information Processing, Biometry, and Epidemiology (IBE), Faculty of Medicine, Ludwig Maximilian University, Munich, Germany; 3 Department of Sports and Health Sciences, Technical University of Munich, Munich, Germany; 4 Institute of Health Economics and Health Care Management, Helmholtz Zentrum München, Munich, Germany; 5 Heidelberg Institute of Global Health, Heidelberg University, Heidelberg, Germany; 6 Department of Health Management and Health Economics, University of Oslo, Oslo, Norway; 7 German Center for Infection Research (DZIF), Partner Site, Munich, Germany; 8 Massachusetts General Hospital/Harvard Medical School, Boston, MA USA; 9 Institute of Social Medicine, Epidemiology and Health Economics, Charité – Universitätsmedizin Berlin, Berlin, Germany

**Keywords:** Cost effectiveness, Diagnostics, Low- and middle-income countries, Point of care, Early infant diagnosis, Health systems

## Abstract

**Background:**

Continuing progress in the global pediatric human immunodeficiency virus (HIV) response depends on timely identification and care of infants with HIV. As countries scale-out improvements to HIV early infant diagnosis (EID), economic evaluations are needed to inform program design and implementation. This scoping review aimed to summarize the available evidence and discuss practical implications of cost and cost-effectiveness analyses of HIV EID.

**Methods:**

We systematically searched bibliographic databases (Embase, MEDLINE and EconLit) and grey literature for economic analyses of HIV EID in low- and middle-income countries published between January 2008 and June 2021. We extracted data on unit costs, cost savings, and incremental cost-effectiveness ratios as well as outcomes related to health and the HIV EID care process and summarized results in narrative and tabular formats. We converted unit costs to 2021 USD for easier comparison of costs across studies.

**Results:**

After title and abstract screening of 1278 records and full-text review of 99 records, we included 29 studies: 17 cost analyses and 12 model-based cost-effectiveness analyses. Unit costs were 21.46–51.80 USD for point-of-care EID tests and 16.21–42.73 USD for laboratory-based EID tests. All cost-effectiveness analyses stated at least one of the interventions evaluated to be cost-effective. Most studies reported costs of EID testing strategies; however, few studies assessed the same intervention or reported costs in the same way, making comparison of costs across studies challenging. Limited data availability of context-appropriate costs and outcomes of children with HIV as well as structural heterogeneity of cost-effectiveness modelling studies limits generalizability of economic analyses of HIV EID.

**Conclusions:**

The available cost and cost-effectiveness evidence for EID of HIV, while not directly comparable across studies, covers a broad range of interventions and suggests most interventions designed to improve EID are cost-effective or cost-saving. Further studies capturing costs and benefits of EID services as they are delivered in real-world settings are needed.

**Graphical Abstract:**

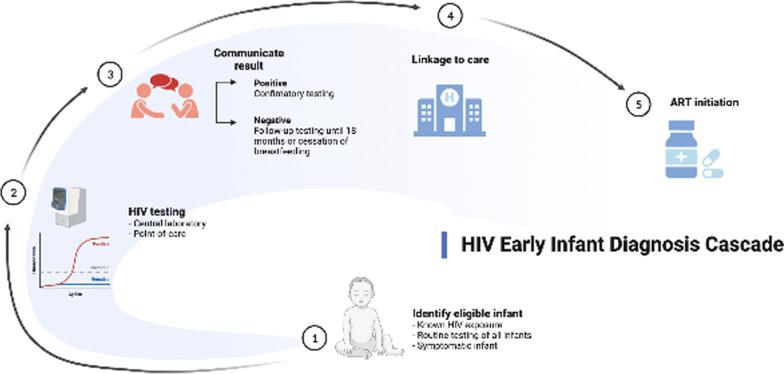

**Supplementary Information:**

The online version contains supplementary material available at 10.1186/s40249-022-01006-7.

## Background

Approximately 1.3 million infants are exposed to human immunodeficiency virus (HIV) each year through gestation, childbirth, and breastfeeding [1]. Despite tremendous global progress in expanding prevention of mother-to-child transmission (PMTCT) services, an estimated 150,000 children were newly infected with HIV in 2020 [2]. Approximately half of new infections occur during gestation and childbirth [3]. Disease progression among infants with HIV is rapid with mortality peaking in the first 2–3 months of life [4] and reaching 50% after 2 years [5]. Early diagnosis and antiretroviral treatment (ART) can significantly improve survival [6–8] and thus are critical to continue global pediatric HIV progress.

Conventional early infant diagnosis (EID), typically performed by centralized laboratories, is logistically complex. It requires caregivers to return to the health facility with their infants several times after delivery to initiate testing, receive results, complete follow-up testing, and initiate care. Despite substantial recent investment in diagnostic networks and centralized laboratory capacity, only 63% of HIV-exposed infants received an EID test by the recommended 4–8 weeks of age in 2020 [9, 10]. Further, nearly 40% are no longer in care by 18 months of age, with most loss to follow-up occurring in the first 6 months [11]. While conventional, central laboratory-based EID programs can reduce costs through economies of scale, this approach results in frequent diagnostic delays and loss to follow-up, limiting access to ART. Only 54% of children living with HIV received ART in 2020 [12].

Several strategies have been assessed to improve existing EID services and thus the health and survival of HIV-exposed infants. Point-of-care (PoC) testing improves turnaround times from sample collection to communication of results and ART initiation [13–17] and is recommended by the World Health Organization (WHO) [18]. Other interventions aimed at reducing turnaround time of conventional, laboratory-based testing, such as SMS printers, mobile/electronic health solutions, more efficient sample transport, and the use of hub-and-spoke models for EID have been evaluated on a limited basis in LMICs [19–23]. Adding HIV testing at birth offers potential to improve EID coverage and reduce pre-ART mortality through earlier identification and treatment of infants with HIV [24, 25]. Expanding access to EID beyond PMTCT programs offers the opportunity to identify infants who may be missed by conventional EID programs, especially in settings with high maternal HIV prevalence and low coverage of PMTCT services [26]. Further integrating HIV care for mothers and infants by providing combined interventions from the continuum of health and social services (e.g., adherence support, assisted disclosure of HIV status) as well as engaging the community in the delivery of health services (e.g., mentor mothers) can increase coverage, engagement in care, cost-effectiveness, and sustainability [27, 28].

Evidence of success of EID interventions identifying infants with HIV, improving linkage to care, demonstrating operational feasibility, and improving overall patient outcomes is accumulating [13–15, 17, 29]. However, limited evidence on the economic implications of these interventions is available. To inform decisions about EID program design and implementation, costs and cost-effectiveness estimates of EID are needed, particularly for high HIV burden, resource-poor settings. In this scoping review, we systematically summarize the available literature on the costs and cost-effectiveness of EID in low- and middle-income countries (LMICs). We also discuss practical implications and key limitations of existing studies.

## Methods

We conducted a scoping review, following the Preferred Reporting Items for Systematic Reviews and Meta-Analyses extension for Scoping Reviews (PRISMA-ScR) checklist [30] as well as general related guidance [31]. A study protocol was made publicly available on the Open Science Framework on June 8, 2021 [27]. In line with PRISMA-ScR recommendations, we did not perform a quality appraisal of the included studies.

### Information sources and search strategy

We searched the bibliographic databases Embase and MEDLINE (via Ovid) and EconLit (via EBSCOhost) for eligible pre-print and peer-reviewed records published in English between January 1, 2008 and June 8, 2021. We restricted our search to records published since 2008 based on 2008 WHO guidance recommending all HIV-exposed infants be tested by 2 months of age followed by immediate ART initiation for infants with HIV [32]. We also searched the archives of major HIV conferences (International AIDS Society conferences including AIDS and the Conference on Retroviruses and Opportunistic Infections) and Google Scholar (stopping screening after 50 irrelevant hits). The search strategy was based on four search terms clusters: HIV, infants, EID, and costs/cost-effectiveness (Additional file 1: Table S1).

### Inclusion and exclusion criteria

We included studies of HIV-exposed infants in LMICs (defined by the World Bank classification [33]) exposed to interventions/programs aimed at improving access to EID and/or completion of the EID cascade [34] and reporting costs or cost-effectiveness outcomes. The EID cascade was defined as (1) identification of the HIV-exposed infant (known HIV exposure or symptomatic infant), (2) HIV testing, (3) communication of results, (4) linkage to care, and (5) ART initiation. If applicable, relevant comparators were alternative interventions or the local standard-of-care. We excluded commentaries, correspondence articles, and reviews, but screened the references of reviews to identify additional original articles for inclusion.

### Outcomes

Our primary extracted outcomes were reported costs, cost savings, incremental cost-effectiveness ratios (ICERs), and net health or monetary benefit, as defined by Drummond et al. [35]. Secondary outcomes related to health or the EID care process (e.g., turnaround time, proportions of infants initiating treatment) were extracted as alternative disease-specific effects to enrich our discussion of the economic evidence for EID within the context of LMIC infant populations where health utilities are typically unavailable. For articles that did not report the reference year for costs, we assumed it to be 2 years prior to publication. All costs were converted to 2021 USD using the International Monetary Fund Gross Domestic Product (GDP) annual deflator for the United States [36].

### Study screening, data extraction and analysis

Three review authors (KE, KEF, BPG) screened titles and abstracts of retrieved records after the removal of duplicates using Covidence [37]. Full-text review was conducted by KE, AE, and VO. Two review authors (AE and VO) extracted outcome data from the included studies to Microsoft Excel 16.60 (Microsoft Corporation, Redmond, USA), and a third review author (KE) cross-checked the data. Discrepancies were discussed among the authors and resolved by consensus. Extracted data were summarized in narrative and tabular formats. Descriptive statistics including frequencies and percentages and ranges of costs for comparable tests were compiled.

## Results and discussion

### Characteristics and data sources of included studies

**Fig. 1:**
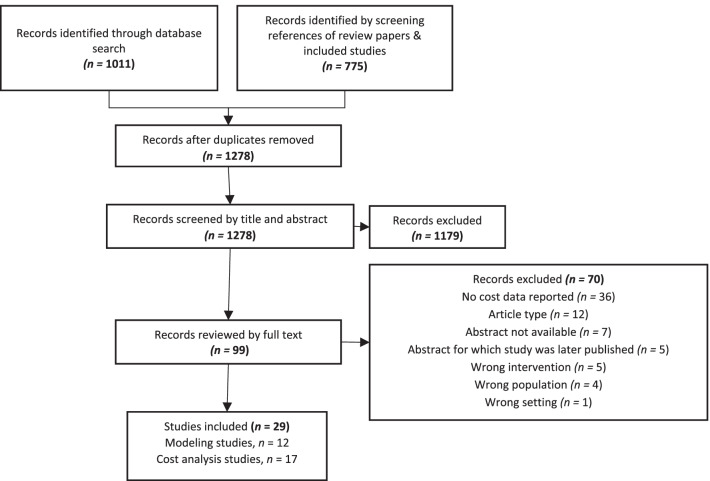
PRISMA flow diagram

We identified 1786 studies including 1011 studies from database searches and 775 studies from the references of reviews. After removing 508 duplicates, we screened titles and abstracts of 1278 studies and reviewed the full text of 99 studies. We included 29 studies on the costs and cost-effectiveness of EID. Reasons for exclusion were lack of cost data (51%), article type (e.g., review or opinion article) (17%), unavailability of abstract (10%) or abstract for which the full results were later published (7.1%), interventions not related to EID (7.1%), population not HIV-exposed infants (5.7%), or study setting outside of LMICs (1.4%) (Fig. 1).

Among the included studies, there were 12 model-based cost-effectiveness analyses (11 full texts and one abstract) and 17 cost analyses (14 full texts and three abstracts) published between 2008 and 2021 (Additional file 1: Table S3). All studies were conducted in sub-Saharan Africa except for one study from Thailand [38]. Nine cost analyses included primary cost data collection [39–47]. Cost estimates for other studies were derived from programmatic data, published estimates (e.g., Clinton Health Access Initiative, Global Fund [48, 49]), or the literature. Effectiveness data used in cost-effectiveness analyses were collected from Joint United Nations Programme on HIV/AIDS pooled analyses, WHO and UNICEF estimates, programmatic data, and the literature.

### Costs per HIV early infant diagnosis test

We categorized EID tests into four groups: PoC-nucleic acid testing (NAT; e.g., Abbott m-PIMA, Cepheid GeneXpert®), laboratory-based NAT, rapid antigen- or antibody-based tests, and unspecified NAT. Currently, only NAT are recommended for EID [18]. Unit costs per test are reported in Table 1. Seven studies reported unit costs for PoC assays [13, 40, 50–54], 13 for laboratory testing [13, 24, 38, 39, 41, 44, 46, 50–55], two for rapid testing [41, 56], and one for unspecified NAT [57]. All unit costs are expressed in 2021 USD unless otherwise specified. Reported PoC-NAT cost per test were 21.46–51.80 USD. Costs for commercially available laboratory-based NAT were 16.21–42.73 USD.

**Table 1:** Unit cost per test for HIV early infant diagnosis

Test	Reported unit cost (USD) per test (range)	Currency of reported unit cost	Converted (USD 2021) unit cost per test	Services included in the unit cost	Setting	Source of cost data	Source(s)
**PoC nucleic acid testing**							
m-PIMA	25 (23–27)	2018 USD	26.82 (24.68–28.97)	Reagents, sample collection, labor	sub-Saharan African countries	CHAI	Salvatore et al. [50]
m-PIMA (reagent rental model^a^)	25.89	2017 USD	28.44	Reagents, blood collection, freight (insurance and customs clearance), training, facility upgrades, site monitoring, labor, error rate^b^	Zimbabwe	Financial records and other secondary sources	Mukherjee et al. [40]
m-PIMA	44.55	2017 USD	48.94	Platform and warranty purchase, reagents, blood collection, freight (insurance and customs clearance), storage and distribution, training, facility upgrades, site monitoring, labor, error rate^b^			
m-PIMA	48.28	2018 USD	51.80	Platform purchase and install, maintenance, freight, and distribution; reagents, blood collection, waste management, labor	Zambia	NSEBA study, CHAI	De Broucker et al. [53]
m-PIMA or GeneXpert IV	27.24 (21.39–33.10) optimal throughput 37.89 (32.54–43.25) current throughput^b^	USD, year not specified	29.93 (23.50–36.36) 40.65 (34.91–46.41)	Reagents, controls, and other consumables, and apportioned costs of equipment, logistics, training, service, and maintenance	Cameroon, Côte d’Ivoire, Kenya, Lesotho, Mozambique, Rwanda, Swaziland, and Zimbabwe	The Global Fund	Bianchi et al. [13]
GeneXpert IV & GeneXpert Edge	20 (18–22)	2018 USD	21.46 (19.31–23.61)	Reagents, sample collection, waste management, labor	sub-Saharan African countries	CHAI	Salvatore et al. [50]
GeneXpert IV (no equipment costs)	23.85	2017 USD	26.20	Reagents, sample collection, waste management, freight (insurance and customs clearance), storage and distribution, training, facility upgrades, site monitoring, labor, error rate	Zimbabwe	Financial records and other secondary sources	Mukherjee et al. [40]
GeneXpert IV Gel	27.27		29.96	Same as above + platform and warranty purchase, gel battery^b^			
GeneXpert IV Solar	27.70		30.43	Same as above + platform and warranty purchase, solar battery^b^			
GeneXpert IV Gel	30.71	2017 USD	33.74	Same as Mukherjee 2020 GeneXpert IV Gel	Zimbabwe	Mukherjee 2020	McCann et al. [51]
GeneXpert	27.91	2018 USD	29.95	Platform purchase and install, maintenance, freight, and distribution; reagents, blood collection, waste management, labor	Zambia	NSEBA study, CHAI	De Broucker et al. [53]
Unspecified PoC EID assay	27.61	2016 USD	30.91	Reagents, controls, and other consumables, and apportioned costs of equipment, logistics, training, service, and maintenance	Zimbabwe	The Global Fund	Frank et al. [52]
Unspecified PoC EID assay	30	2013 USD	34.90	Not specified	South Africa	Assumption	Dunning et al. [54]
**Laboratory-based nucleic acid testing**							
Roche COBAS Ampliprep®/TaqMan®	15.11	2018 USD	16.21	Reagents, sample collection, transport, waste management, labor	sub-Saharan Africa	CHAI	Salvatore et al. [50]
Abbott m2000	17.41	2018 USD	18.68				
Laboratory-based NAT (LAB)	18.10	2017 USD	19.89	Not specified	Zimbabwe	Nichols 2019	McCann et al. [51]
Strengthened laboratory-based NAT (S-LAB)	30.47	2017 USD	33.48	Same as above + daily sample transport, EID-specialized personnel, additional training		EGPAF programmatic data	
Roche Amplicor HIV-1 DNA PCR	21.50	USD, year not specified	26.46	Insurance, freight and tax charges, filter paper, reagents, courier service, labor	Kenya	Not listed	Khamadi et al. [39]
Unspecified laboratory-based NAT	25	2013 USD	29.08	Not specified	South Africa	NHLS South Africa—personal communication	Dunning et al. [54]
Unspecified laboratory-based NAT	25	2013 USD	29.08	Assays, reagents, personnel time for counseling, blood collection, specimen transport and processing, quality control	South Africa	Assumption	Franke et al. [24]
Roche Amplicor HIV-1 DNA rtPCR v1.5	23.32–23.76	2007 USD	29.80–30.36	Equipment, assay, sample collection, labor	Uganda	Data collected during study	Menzies et al. [41]
Unspecified laboratory-based NAT	27.61	2016 USD	30.91	Reagents, controls, and other consumables, and apportioned costs of equipment, logistics, training, service, and maintenance	Zimbabwe	The Global Fund	Frank et al. [52]
Unspecified DNA-rtPCR	32.40	2017 USD	35.60	Not specified	Tanzania	Hospital data	Vyas et al. [46]
Roche COBAS Ampliprep®/TaqMan®	38.07	2018 USD	40.85	Platform purchase and install, maintenance, freight, and distribution^b^; reagents, blood collection, waste management, labor	Zambia	NSEBA study, CHAI	De Broucker et al. [53]
Roche or Abbott conventional rtPCR	38.89 (28.57–49.21) result within 3 months 131.02 (96.26–165.76) result within 30 days	USD, year not specified	42.73 (31.39–54.06) 143.94 (105.75–182.11)	Reagents, controls, and other consumables, and apportioned costs of equipment, logistics, training, service, and maintenance	Cameroon, Côte d’Ivoire, Kenya, Lesotho, Mozambique, Rwanda, Swaziland, and Zimbabwe	The Global Fund	Bianchi et al. [13]
Unspecified laboratory-based NAT	40.50	2016 USD	45.34	Sample collection, counseling, transport, laboratory test costs	Lesotho	Study data	Tchuenche et al. [44]
Proviral DNA rtPCR in-house assay from DBS	8–10	USD, year not specified	9.05–11.31	Filter paper, reagents, equipment maintenance, human resources	Angola	Data collected during study	Martin et al. [55]
DNA rtPCR in-house assay from DBS	57.14	2011 USD	68.90	Equipment, reagents, blood collection, transport, labor, maintenance	Thailand	Sirirungsi (2013); Clinton Foundation (2009)	Collins et al. [38]
**Rapid HIV test**							
PoC p24 antigen detection test	< 15 per assay	USD, year not specified	15.82	Not specified	Zambia	Study data	Sutcliffe et al. [56]
Initial Rapid RHT + confirmatory PCR for positive infants	7.58–22.75^c^	2007 USD	9.68–29.07	Assay, sample collection, labor	Uganda	Study data	Menzies et al. [41]
**Unspecified NAT**							
NAT per local EID programs	24	2018 USD	25.75	Not specified	Cote d'Ivoire, South Africa, Zimbabwe	The Global Fund	Dunning et al. [57]

*USD* United States dollar, PoC point-of-care, *CHAI* Clinton Health Access Initiative, *EID* early infant diagnosis, *rtPCR* reverse transcriptase polymerase chain reaction, *DBS* dried blood sample, *NAT* nucleic acid testing

^a^Consolidated cost for testing cartridges inclusive of equipment, maintenance, data, and connectivity, assuming 1300 tests/platform/year and including VL assays, over 3 years

^b^Incorporates utilization (i.e., the ability of the machine to run additional assays including HIV viral load for mPIMA and HIV viral load and tuberculosis for GeneXpert®)

^c^Range dependent on infant age and symptoms. Cost includes RHT + confirmatory DNA-PCR if RHT is positive. Rapid RHT activity cost is 0.88 USD

Variation in unit costs for PoC assays may be explained by inclusion of capital costs. Costs for m-PIMA that included equipment costs were > 20 USD higher than those that did not include equipment costs. GeneXpert® costs per test were less sensitive to variation due to inclusion of equipment costs, with studies excluding equipment costs reporting 21.46–26.20 USD and those including equipment costs reporting 29.96–33.74 USD (Table 1). This may be due to the incorporation of utilization, including the ability to run multiple tests simultaneously, in the unit cost. m-PIMA can run one test at a time, whereas GeneXpert® analyzers support two or four tests run simultaneously. However, utilization is only relevant when equipment costs are included and only one study including equipment costs from Zimbabwe specified that they considered utilization [40]. The types of services that were included in the unit costs for centralized laboratory testing were more varied and less commonly detailed.

One study, conducted in Zambia, evaluated PoC p24 assays which may be more affordable than PoC-NAT tests (< 15 USD per test) and do not require specialized equipment [56]. Despite low sensitivity in very young infants, PoC p24 assays could play a role in diagnosing infants > 4 weeks of age at rural sites where the significant capital investment in PoC-NAT testing platforms is not feasible [56]. An assay that is 80% sensitive and links 99% of positive infants to care achieves the same level of ART coverage as an assay that is 95% sensitive and only links 85% of positive infants to care [58]. However, PoC p24 assays are currently not approved.

A study from Uganda reported that rapid antibody screening before EID testing of infants with a positive serology result was a cost-saving measure at 10–30 USD per test [41]. This is no longer recommended in the context of declining MTCT rates as well as wider availability of NAT and inferior sensitivity of antibody tests compared to NAT [18].

### HIV early infant diagnosis program costs

Among 24 studies reporting costs of an EID intervention or program, these were reported as lifetime cost per HIV-exposed infant, average cost per HIV-positive diagnosis, cost per HIV-exposed infant person-year, or total price of the intervention/program (Table 2). Few studies assessed the same intervention or reported costs in the same way, making comparison of costs across studies challenging. Most studies evaluated costs or cost-effectiveness of EID testing approaches including four studies on PoC EID [45, 50, 52, 53], three on birth testing [24, 44, 59], one study that assessed both PoC and improvements to centralized EID [51], one that reported costs of confirmatory testing in EID programs [54], one of added screening of mothers at 6-week infant immunization visits with referral to EID for infants at risk of acquiring HIV [57], and one of rapid antibody screening to rule out negative infants before NAT [41].

**Table 2:** Costs of early infant HIV diagnosis interventions and programs

Intervention	Total reported cost of intervention (USD)	Currency of reported cost	Total converted cost of intervention (USD 2021)	Description	Setting	Source(s)
**Testing strategies—per person costs**						
NAT (lab) at birth + 6 weeks	870/HIV-exposed infant	2013 USD	1012	Discounted cost/infant including EID, ART, routine care and monitoring, opportunistic infections, and death	South Africa	Franke et al. [24]
NAT (lab) at 6 weeks only	820/HIV-exposed infant		954			
Birth + 6-week EID testing	1379/HIV-exposed infant	USD, year not specified	1632	Cost per HIV-infected diagnosis	South Africa	Collins et al. [59]
6-week EID testing only	458/HIV-exposed infant		542			
Total incremental cost of adding NAT at birth^a^	8060/HIV-infected diagnosis	2015 USD	9114	Cost (clinical labor, drugs, supplies, commodities, support staff, construction and renovation, equipment, sample transport) per early infection identified and started on ART	Lesotho	Tchuenche et al. [44]
PoC EID (GeneXpert Gel)	240/HIV-exposed infant	2017 USD	264	Discounted HIV-related lifetime costs including PoC strategy costs, HIV care, and ART	Zimbabwe	McCann et al. [51]
PoC EID (platform not specified)	420/HIV-exposed infant	2016 USD	470	Discounted EID testing costs for 6-week testing, HIV-related lifetime costs including HIV care, CD4 test, VL test, ART regimen costs	Zimbabwe	Frank et al. [52]
Strengthened laboratory-based EID (S-LAB)	222/HIV-exposed infant	2017 USD	244	Discounted HIV-related lifetime costs including HIV care, strengthened laboratory-based strategy costs, and ART	Zimbabwe	McCann et al. [51]
Testing at 6 weeks, with confirmatory testing	1790/HIV-exposed infant tested	2013 USD	2082	Lifetime cost per HIV-exposed infant including cost of NAT and return of results, routine HIV care, ART, opportunistic infection care, and major toxicity events	South Africa	Dunning et al. [54]
Testing at 6 weeks, without confirmatory testing	1830/HIV-exposed infant tested		2129			
Universal maternal HIV screening at infant immunization visits with referral to EID	1. 60/mother-infant pair 2. 180/mother-infant pair 3. 100/mother-infant pair	2018 USD	1. 64 2. 193 3. 107	Screen-and-test per-person lifetime costs including maternal HIV screening, infant NAT, routine HIV care, acute OI care, and pediatric ART	1. Cote d'Ivoire 2. South Africa 3. Zimbabwe	Dunning et al. [57]
Initial rapid RHT testing to screen-out HIV-uninfected infants before DNA-rtPCR	147 (average cost per HIV positive infant correctly diagnosed and informed of result)	2007 USD	188	Testing activity costs including personnel and supplies for pre-test counseling, sample collection and preparation, rapid HIV testing, DNA-PCR testing, and post-test counseling	Uganda	Menzies et al. [41]
**Testing strategies—per population costs**						
PoC testing (m-PIMA) including confirmatory test	4,246,527 (total program costs)	2018 USD	4,556,354	Capital costs including platform purchase, installation, insurance, and maintenance, sample transport, and training. Recurrent costs including reagents, blood collection supplies, and staff time for testing up to three times (birth, 6 weeks, and 6 months	Zambia	De Broucker et al. [53]
PoC testing (GeneXpert) including confirmatory test	2,851,894 (total program costs)		3,059,969			
PoC testing (m-PIMA) with confirmatory testing in central laboratory	4,339,757 (total program costs)		4,656,387			
PoC testing (GeneXpert) with confirmatory testing in central laboratory	2,945,768 (total program costs)		3,160,692			
PoC testing (m-PIMA) 1. Low PMTCT setting 2. High PMTCT setting	Total EID program costs 1. 1,818,000 2. 1,801,000	2018 USD	1. 1,950,642 2. 1,932,401	Capital costs including service and maintenance, freight, insurance, inspection, handling, and customer service delivery. Recurrent costs including reagents, consumables, sample collection, transport, and waste management	sub-Saharan Africa	Salvatore et al. [50]
PoC testing (GeneXpert) 1. Low PMTCT setting 2. High PMTCT setting	Total EID program costs 1. 1,662,000 2. 1,647,000		1. 1,783,260 2. 1,767,166			
PoC testing (GeneXpert) Edge 1. Low PMTCT setting 2. High PMTCT setting	Total EID program costs 1. 1,148,000 2. 1,134,000		1. 1,231,758 2. 1,216,737			
PoC (m-PIMA) + centralized testing 1. Low PMTCT setting 2. High PMTCT setting	Total EID program costs 1. 1,818,000 2. 1,802,000		1. 1,950,642 2. 1,933,474			
PoC (GeneXpert) + centralized testing 1. Low PMTCT setting 2. High PMTCT setting	Total EID program costs 1. 1,662,000 2. 1,648,000		1. 1,783,260 2. 1,768,238			
PoC (GeneXpert Edge) + centralized testing 1. Low PMTCT setting 2. High PMTCT setting	Total EID program costs 1. 1,148,000 2. 1,134,000		1. 1,231,758 2. 1,216,737			
PoC (GeneXpert)	31,695 total implementation cost	2019 USD	33,410	Infrastructure, PoC testing, maintenance and repairs during study, training, labor including travel and accommodation	Rural Zambia	Sutcliffe et al. [45]
**Other interventions**						
Sample transfer model	1. 20–40 2. 4,244,000	USD, year not specified	1. 23.27–46.53 2. 5,117,496	Sample transfer per batch Not listed	1. Nigeria 2. Uganda	1. Ndulue et al. [60] 2. Kiyaga et al. [22]
1. Single well-equipped and staffed lab for EID 2. Four-lab EID system 3. Eight-lab EID system	Total cost not listed, see description	USD, year not specified	N/A	1. Reagents (5,076,035), consumables (122,276), DBS collection supplies (1,015,834), transport to districts (476,024), recurrent costs (2,821,761) 2. Reagents (5,076,035), consumables (122,276), DBS collection supplies (1,015,834), transport to districts (457,944), recurrent costs (4,593,200) 3. Reagents (3,893,435), consumables (923,510), DBS collection supplies (1,015,834), transport to districts (433,844), recurrent costs (6,960,344)	Uganda	Kiyaga et al. [61]
Expedited results system (ERS) with GPRS	0.0002/result transmitted	USD, year not specified	0.0003	Cost of transmitting each result using GPRS technology	Mozambique	Jani et al. [23]
HITSystem (infant tracking system)	Total cost not listed, see description	USD, year not specified	N/A	1. Direct implementation costs/month/hospital (mobile broadband minutes, patient tracing, texting, data storage): 350. One-time start-up costs/hospital (training, quality assurance, computer and modem purchase): 100–400 2. Fixed monthly costs include a 200 SMS and secure data storage fee and ~ 50 for mobile broadband minutes	Kenya	1. Finocchiaro-Kessler et al. [62] 2. Finocchiaro-Kessler et al. [63]
Mobile phone follow-up for EID services	0.76	USD, year not specified	0.94	Average cost per HIV-exposed infant returned to care	Uganda	Kiyaga et al. [20]
Quality assurance system (QAS)	Kenya: 208,532/year South Africa: 69,359/year Senegal: 102,853/year Uganda: 203,330/year Zimbabwe: 334,342/year	2016 USD	Kenya: 233,432/year South Africa: 77,641/year Senegal: 115,134/year Uganda: 227,609/year Zimbabwe: 374,265/year	Total and average annual quality assurance system costs including start-up costs, capital costs, recurrent costs including a 10% wastage rate for supplies, and corrective action costs	Kenya, Senegal, South Africa, Uganda, Zimbabwe	Terris-Prestholt et al. [43]
Centralized EID with deferred ART based on immune/clinical criteria	5,254,683/all children	2011 USD	6,336,196	Pre and post HIV test counselling, HIV diagnosis, ART	Thailand	Collins et al. [38]
Centralized EID with immediate ART	6,773,115/all children		8,167,151			
Co-located MCH care throughout breastfeeding	14,674/HIV-infected infant	2016 USD	16,426	Lifetime cost for all HIV-infected children in this system	South Africa	Dugdale et al. [27]
Separate ART services for mothers and infants, referral post-delivery	14,617/HIV-infected infant		16,362			
Neonatal HIV care (Nevirapine + DNA-PCR at 6 weeks)	90.09/HIV-exposed infant	2017 USD	98.98	DNA-PCR, other supplies, utilities, Nevirapine, capital costs including building, equipment, and training,	Tanzania	Vyas et al. [46]
EID program (testing approach unspecified)	1. 60.92/infant tested 2. 10.91/infant tested	2009 USD	1. 75.89 2. 13.59	Nurse, laboratory technician, driver, reagents, miscellaneous items	1. Namibia 2. Rwanda	Touré et al. [42]
EID services (not specified)	1. 28.04/PPY HIV-exposed infant 2. 12.08/PPY HIV-exposed infant	2014 USD	1. 32.02 2. 13.79	Not specified	Ethiopia	Zegeye et al. [47]

*USD* United States dollar, *PoC* point-of-care, *EID* early infant diagnosis, *rtPCR* reverse transcriptase polymerase chain reaction, *DBS* dried blood sample, *NAT* nucleic acid test, *MCH* maternal and child health, *PPY* per person-year

^a^Assuming 66.3% of infants whose mothers are accessing PMTCT services are tested

Lifetime PoC EID testing costs were estimated at 264 and 470 USD per infant in Zimbabwe [51, 52] and 1.2–4.7 million USD total program costs [50, 53] in representative sub-Saharan African countries. Modelled total PoC EID program costs were slightly higher for m-PIMA compared to GeneXpert® but similar for settings with low and high PMTCT coverage [50, 53]. While unit costs for PoC EID are generally higher than laboratory-based testing, PoC testing addresses well-recognized challenges of conventional laboratory-based EID including improving turnaround times, increasing the proportion of infants with HIV initiating ART, and leading to earlier ART initiation [13, 15, 17, 29]. As initial investment in PoC-NAT platforms and infrastructure to support decentralized testing is significant [45], costs are highly impacted by throughput. Average throughput across eight sub-Saharan African countries in a 2019 study was 0.7–3 tests/day/health facility with an associated additional cost of 10 USD/test compared with optimal throughput (defined as 70% of platform capacity) in the same setting [13]. Integrating capital costs across programs (e.g., HIV viral load and tuberculosis testing) and/or health facilities via hub-and-spoke models and thereby increasing throughput can reduce costs [50]. Similarly, personnel sharing across services may increase efficiency without lowering the quality of services [46].

The discounted cost of birth testing from a modelling study in South Africa was 1012 USD per HIV-exposed infant with an *in-utero* infection rate of 1.8% [24]. The incremental cost of testing infants exposed to HIV at birth in Lesotho was 9114 USD per infant identified as infected at birth with an *in-utero* infection rate of 0.5%. This decreased to 2289 USD with an *in-utero* infection rate of 2%, similar to the undiscounted cost of 2140 USD per infant in the previous study. In countries with low coverage of PMTCT programs and higher *in-utero* infection rates (e.g., Nigeria [1, 2]) birth testing may be cost-effective compared to birth plus 6-week testing [44]. Targeted testing at birth only for infants at elevated risk of HIV acquisition (e.g., mother started ART late in pregnancy or has a high viral load around the time of delivery) reduces the burden on an already strained health workforce and therefore may be more appropriate for settings with low *in-utero* transmission rates [44].

Studies of other service delivery interventions, including co-located post-partum maternal and child health services in South Africa [27], sample transport in Uganda and Nigeria [22, 60], consolidation of EID testing in a single lab in Uganda [61], electronic communication systems in Uganda, Mozambique, and Kenya [20, 23, 62, 63], and a quality assurance system modelled in five sub-Saharan African countries [43], were also identified (Table 2). One study reported costs of immediate versus delayed ART initiation following EID testing in Thailand [38]. Three studies focused on cost variations across region or type of health facility within existing programs [42, 46, 47]. These studies reported wide variation of cost estimates across settings and therefore recommended context-specific cost estimates to inform budgeting and planning [46].

### Cost-effectiveness of HIV early infant diagnosis

Table 3 summarizes the results of the 12 cost-effectiveness analyses. All studies stated at least one of the interventions evaluated to be cost-effective or cost-saving. ICERs were expressed as incremental costs per year-of-life saved (YLS)/per life-years gained (LYG), per death averted, or per additional infant initiating ART within 60 days. One study modelled costs and effects separately and did not report an ICER [59] and one study only reported an ICER for mother-infant pairs [27], and these were not included in the table, however costs were included in Tables 1 and 2.

**Table 3:** Cost-effectiveness analysis of HIV early infant diagnosis results of included studies

Intervention	Comparator	ICER (USD)	Setting	Currency	Willingness to pay threshold (USD)	Evidence of cost-effectiveness	Source
NAT at 6 weeks only	No EID testing strategy	1250/YLS	South Africa	2013 USD	50% of GDP (3416). Also examined thresholds of 100% and 300% of GDP	Yes	Francke et al. [24]
NAT at birth + 6 weeks	NAT at 6 weeks only	2900/YLS					
PoC EID	SoC: conventional laboratory-based EID	680/YLS	Zimbabwe	2016 USD	1 × GDP (1010)	Yes	Frank et al. [52]
PoC EID (GeneXpert Gel)	SoC: conventional laboratory-based EID	830/YLS	Zimbabwe	2017 USD	1. 1 × GDP (1600/YLS) 2. 1 × lifetime ART regimen (580/YLS)	1. Yes	McCann et al. [51]
Strengthened laboratory-based EID		Dominated				2. No	
PoC testing (mPIMA)	SoC: conventional laboratory-based testing (COBAS AmpliPrep®/TaqMan®)	1554/additional infant on ART within 60 days 5976/death averted	Zambia	2018 USD	Not listed	Yes	De Broucker et al. [53]
PoC testing (GeneXpert)		23/additional infant on ART within 60 days 90/death averted					
PoC testing (mPIMA)	SoC: Centralized testing		sub-Saharan Africa	2018 USD	Not listed	Yes	Salvatore et al. [50]
1. Low PMTCT setting		1. 1475/death averted					
2. High PMTCT setting		2. 3888/death averted					
PoC testing (GeneXpert)							
1. Low PMTCT setting		1. 1297/death averted					
2. High PMTCT setting		2. 3426/death averted					
PoC testing (GeneXpert Edge)							
1. Low PMTCT setting		1. 591/death averted					
2. High PMTCT setting		2. 1527/death averted					
PoC testing (mPIMA) + central testing							
1. Low PMTCT setting		1. 1507/death averted					
2. High PMTCT setting		2. 3963/death averted					
PoC testing (GeneXpert) + central testing							
1. Low PMTCT setting		1. 1357/death averted					
2. High PMTCT setting		2. 3574/death averted					
PoC testing (GeneXpert Edge) + central testing							
1. Low PMTCT setting		1. 618/death averted					
2. High PMTCT setting		2. 1593/death averted					
Testing at 6 weeks, with confirmatory testing	Testing at 6 weeks, without confirmatory testing	Cost-saving	South Africa	2013 USD	Not Listed	Yes	Dunning et al. [54]
Initial rapid HIV testing to screen-out HIV-uninfected infants before DNA-rtPCR	DNA-rtPCR with Roche Amplicor v1.5	1489/infant correctly diagnosed and informed of result	Uganda	2007 USD	Not listed	Yes	Menzies et al. [41]
Universal HIV exposure screening at infant immunization visits with referral to EID	SoC: 6-week NAT for infants with known HIV exposure	1. 1340/YLS	1. Cote d'Ivoire	2018 USD	1 × GDP (1720/6380/2150, respectively)	Yes	Dunning et al. [57]
		2. 650/YLS	2. South Africa				
		3. 670/YLS	3. Zimbabwe				
Centralized EID with deferred ART based on immune/clinical criteria	Clinical/serology-based diagnosis and deferred ART	5149/LYG	Thailand	2011 USD	1 × GDP (4420)	No	Collins et al. [38]
Centralized EID with immediate ART		2615/LYG				Yes	
Quality assurance system (QAS)	No quality assurance system, misdiagnosis rate 5%	1. Kenya: cost-saving	1. Kenya	2016 USD	1. Kenya: 316,559	Yes	Terris-Prestholt et al. [43]
		2. South Africa: cost-saving	2. Senegal		2. South Africa: 353,251		
		3. Senegal: 107	3. South Africa		3. Senegal: 3949		
		4. Uganda: cost-saving	4. Uganda		4. Uganda: 702,078		
		5. Zimbabwe: cost-saving	5. Zimbabwe		5. Zimbabwe: 656,845		

*ICER* incremental cost-effectiveness ratio; *NAT* nucleic acid testing, *EID* early infant diagnosis of HIV, *YLS* years of life saved, *USD* United States dollar, *GDP* gross domestic product, *PoC* point-of-care, *SoC* standard-of-care, *PMTCT* prevention of mother-to-child-transmission of HIV, *rtPCR* reverse transcriptase polymerase chain reaction, *DBS* dried blood sample, *LYG* life-years gained

Included studies used the Cost-Effectiveness of Preventing AIDS Complications Pediatric model [24, 27, 51, 54, 57, 64] (i.e., a validated state transition model simulating individual costs and HIV disease outcomes [65, 66]), decision tree models [41, 43, 53, 59], and cohort state transition simulation models [38, 50]. Seven studies used a lifetime horizon for the model [24, 27, 38, 51, 52, 54, 57], while the remaining used time horizons of 5 years [53], 2 years [59], 1 year [43], and 18 months [41, 50]. Nine studies used a discount rate of 3% per year for both costs and health benefits [24, 27, 38, 41, 43, 51, 52, 54, 57], two studies reported only undiscounted costs and benefits [50, 53], and one study, an abstract, did not specify whether discounting was applied [59, 67].

Out of four cost-effectiveness studies comparing PoC-NAT to centralized testing, only two reported a willingness-to-pay threshold. Willingness-to-pay thresholds are vital for decision-makers to be able to assess whether resource allocation for an intervention is worth the investment and are often oriented at the country-specific per-capita GDP, particularly in LMIC settings (WHO CHOICE). ICERs per YLS for PoC EID were 52% [51] and 67% [64] of the country-specific (Zimbabwe) per-capita GDP. ICERs for studies that did not report a willingness-to-pay threshold ranged from 23 to 1554 USD per additional child initiating ART within 60 days and 90–5976 per death averted (2018 USD) and were lower for GeneXpert® compared to m-PIMA [50, 53]. Several models assumed 100% EID uptake [51, 52] which excludes the potential costs and benefits of improving access to EID. This assumption favors PoC testing because it is more likely to increase access to EID compared to laboratory-based programs.

While decentralized testing increases access and linkage to ART, it often comes with increased challenges of supply chain management and maintenance. A system-level quality assurance system added to PoC EID programs and aimed at reducing screening interruptions and the misdiagnosis rate was found to be cost-saving in four of five countries modelled [43]. The modelled quality assurance system included external proficiency testing, reports, and corrective action including supervisory visits, equipment maintenance, and refresher trainings. Quality assurance systems can easily be extended to other PoC testing applications and may improve the overall level of service at primary health facilities.

Confirmatory testing was also demonstrated to be cost-saving in South Africa [54], and two cost-effectiveness analyses of PoC testing included scenarios with PoC and laboratory-based confirmatory testing [50, 53]. Without confirmatory testing, more than 10% of infants initiating ART may not actually be HIV-infected in settings with similar MTCT rates to South Africa [54]. ICERs for confirmatory testing at the PoC versus laboratory were slightly more favorable [50, 53], and the WHO now supports PoC testing to confirm positive results [18].

Two cost-effectiveness studies comparing birth plus 6-week testing to 6-week testing only, conducted in South Africa and Lesotho, concluded that cost-effectiveness of birth plus 6-week testing was dependent on prompt ART initiation and the degree to which ART reduces mortality [24, 44]. Birth plus 6-week testing exceeded the willingness-to-pay threshold of 50% of per-capita GDP in South Africa when the added cost was > 7 USD or NAT costs exceeded ~ 36 (2021 USD) [24]. Several estimates included in this review of both PoC and laboratory-based NAT costs in real-world settings exceeded this value [13, 40, 44, 53].

Tracking of infants testing negative at birth to ensure they complete 6-week testing is crucial to detect intrapartum and early breastfeeding transmission. With loss to follow-up rates > 37% between birth and 6-week testing, 1-year survival for infants with HIV in South Africa was lower compared to testing only at 6 weeks of age [24]. Thus, targeted birth testing of infants at high risk of HIV acquisition may be more appropriate given the significant resource investment in testing and tracking of infants to ensure they complete follow-up testing and are linked to care.

ICERs for HIV exposure screening and referral to EID at infant immunization visits compared to standard 6-week NAT ranged from 10 to 78% of country-specific per-capita GDP in three sub-Saharan African countries [57]. Initial rapid HIV testing to screen out uninfected infants before NAT was stated to be cost-effective in Uganda, however, a willingness-to-pay threshold was not specified [41]. The latter is no longer recommended in the context of declining MTCT rates and inferior sensitivity of rapid diagnostic tests compared to NAT, as well as the wider availability and similar cost of PoC-NAT for EID. Rapid diagnostic tests for HIV serology are recommended for diagnosing HIV in children > 18 months [18].

### Knowledge gaps and practical implications

Several gaps in the literature on the cost-effectiveness of EID were identified here. Compared with effectiveness studies, sources of heterogeneity across economic evaluations are more numerous, limiting generalizability of cost-effectiveness results [68]. Cost-effectiveness analyses in this scoping review most commonly compared costs and health benefits of an intervention with current best practice or standard-of-care. Comparison of results across studies is complicated by the fact that standard-of-care is typically not well defined, differs greatly across settings, and is changing rapidly in many countries. Future cost-effectiveness studies will need to carefully consider further changes to these standard-of-care comparisons to accurately guide decision-making.

Lack of data availability in resource-poor settings, both for costs and long-term outcomes of children living with HIV, means model parameters are informed by few estimates from the literature, and it is often necessary to combine data from multiple sources (Table 1 and Additional file 1: Table S2). Cost-effectiveness analyses included in this review made efforts to use the best available data at the time of the study and used sensitivity analyses to compensate for uncertainty, however, the resulting long-term model predictions are still subject to considerable uncertainty. Considering that resource use and opportunity costs are highly context-dependent, decision-makers should focus on the most applicable studies to their settings to effectively distribute resources rather than attempting to synthesize less applicable results from multiple studies. Where generalizable results are unavailable, conducting further economic evaluations could be considered, incorporating local data on costs and where possible, outcomes of children with HIV [64].

Intervention scenarios discussed here generally assume that existing human resources would be sufficient to cover scale-up of EID interventions including task-shifting testing from laboratories to health facilities with PoC EID. This assumption may be unrealistic in settings where uptake of EID is expected to increase. Future economic analyses could include health system constraints by limiting the feasible coverage of interventions to align with current capacity or account for increased human resource costs related to expanding services.

In the absence of available data, the cost-effectiveness modelling of EID presented here does not incorporate additional activities designed to increase uptake, retention in care, and adherence to treatment. This may include traditional service delivery in healthcare settings as well as community health workers and/or mentor mothers. As a result, there remains a limited understanding of the impact of a comprehensive package of services for EID. With many countries moving towards widespread PoC EID, there is an opportunity for economic evaluations to inform priority setting and support the design of optimal service delivery models, but empirical cost data is needed. Evaluations of EID interventions and programs could therefore consider including data collection of real-world implementation costs. Further, full costs of program delivery including outreach should be represented.

Lastly, there were no studies evaluating the costs or cost-effectiveness of routinely offered facility-based testing. As EID is mostly delivered as part of PMTCT services, infants born to mothers receiving inadequate or no PMTCT interventions who are at higher risk of vertical HIV acquisition are also the most likely not to receive a diagnostic test within the first 2 months of life. In settings with high maternal HIV prevalence and poor PMTCT coverage, facility-based testing of infants with unknown HIV status in a range of clinical settings can help close the gap in EID coverage. The yield of positive test results was found to be high for inpatient care and malnutrition clinics in a systematic review of EID testing outside of PMTCT services [69]. More data on the costs and cost-effectiveness of testing infants in specific healthcare settings as a strategy to reduce HIV-related mortality are needed.

### Strengths and limitations

To our knowledge, this is the first review to broadly describe the economic evidence on multiple EID interventions and/or programs. We conducted a broad search of the literature including peer-reviewed and grey literature and extracted extensive information to summarize EID unit costs, intervention costs and cost-effectiveness findings. We also converted findings to a common currency to increase comparability. Limitations of our study include restricting our search to studies published since the 2008 WHO recommendation to test HIV-exposed infants for HIV by 2 months of age, which is, however, the period in which major developments in EID started. Additionally, while we used a broad coverage GDP deflator rather than a consumer price index, it is unclear how the relevant costs in the respective settings have changed since the studies were conducted. Comparison of economic evidence across studies was limited due to heterogeneity of studies in interventions and comparators evaluated, the scope of costs included, as well as assumptions made in terms of model design. Finally, we did not systematically assess the quality of the included studies and potential resulting biases, as is common for scoping reviews.

## Conclusions

The available cost and cost-effectiveness evidence for EID of HIV covers a broad range of interventions and suggests most EID interventions are indeed cost-effective. Few studies reported cost or cost-effectiveness estimates for the same intervention in comparable settings, and resources included in the cost estimates vary widely. Thus, comparison of costs across studies is challenging. Relatively few studies included primary cost data collection, and several report a lack of context- and setting-specific cost data as a limitation. Similarly, cost-effectiveness modelling studies must make assumptions based on limited data both for costs and outcomes of children exposed to HIV.

Increasing uptake and coverage of EID will likely be achieved through a package of services supporting EID service delivery and engagement in care. The scope of studies in this review did not cover the additional costs and benefits outside of EID programs that such comprehensive service delivery would provide. Future cost and cost-effectiveness studies capturing costs and benefits of EID services as they are delivered in real-world settings are needed to support the needs of decision-makers.

## Supplementary Information

**Additional file 1: Table S1.** Search terms used in OVID Medline and Embase. **Table S2.** Search terms used in EconLit and Google Scholar. **Table S3.** Included studies.

## Data Availability

All data generated or analyzed during this study are included in this published article.

## Abbreviations

ART: Antiretroviral treatment
EID: Early infant diagnosis
GDP: Gross domestic product
HIV: Human immunodeficiency virus
ICER: Incremental cost-effectiveness ratio
LMIC: Low- and middle-income countries
LYG: Life-years gained
MTCT: Mother-to-child transmission
NAT: Nucleic acid test
PMTCT: Prevention of mother-to-child transmission
PRISMA-ScR: Preferred Reporting Items for Systematic Reviews and Meta-Analyses extension for Scoping Reviews
PoC: Point-of-care
USD: United States dollar
WHO: World Health Organization
YLS: Years of life saved
