# Supplementary material for: Costs and cost-effectiveness of HIV early infant diagnosis in low- and middle-income countries: a scoping review
Source: Infect Dis Poverty. 2022 Jul 15;11:82. doi: 10.1186/s40249-022-01006-7 (PMC9284833; doi:10.1186/s40249-022-01006-7)
Supplement: Supplementary file 1 — Additional file 1: Table S1. Search terms used in OVID Medline and Embase. Table S2. Search terms used in EconLit and Google Scholar. Table S3. Included studies. [file 40249_2022_1006_MOESM1_ESM.docx]

**Additional file 1**

**Table S1: Search terms used in OVID Medline and Embase**

| **Search cluster** | **Search terms (OVID Medline and Embase)** |
| --- | --- |
| HIV | ((HIV OR human immunodeficiency virus OR AIDS OR acquired immune deficiency syndrome OR acquired immunodeficiency syndrome).ti,ab,kw OR HIV/ OR human immunodeficiency virus/ OR human immunodeficiency virus infection/) AND |
| Infant | ((infant* OR baby OR babies OR newborn* OR neonate* OR neonatal OR birth).ti,ab,kw OR infant/ OR baby/ OR newborn/ OR birth/ OR Infant, Newborn/) AND |
| Early infant diagnosis | ((“early infant diagnosis” OR “early infant HIV diagnosis” OR EID OR diagnosis OR diagnosing OR test or testing).ti,ab,kw) AND |
| Costs/cost-effectiveness | ((cost* OR economic* OR affordab* OR pric* OR expens* OR expenditure* OR budget*).ti,ab,kw OR cost/ or cost effectiveness analysis/ or economic evaluation/ OR Costs and Cost Analysis/) |

Notes: “ti,ab,kw” refers to title, abstract, keyword search; “/” indicates index term; “*” indicates wild card.

**Table S2: Search terms used in EconLit and Google Scholar**

| **Search cluster** | **Search terms (EconLit and Google Scholar)** |
| --- | --- |
| HIV | (HIV OR human immunodeficiency virus OR AIDS OR acquired immune deficiency syndrome OR acquired immunodeficiency syndrome) AND |
| Infant | (infant* OR baby OR babies OR newborn* OR neonate* OR neonatal OR birth) AND |
| Early infant diagnosis | (“early infant diagnosis” OR “early infant HIV diagnosis” OR EID OR HIV test or HIV testing) AND |
| Costs/cost-effectiveness | (cost* OR economic* OR affordab* OR pric* OR expens* OR expenditure* OR budget*) |

**Table S3**: Included studies

| **Source** | **Title** | **Study type** | **Setting** | **Study Perspective** | **Data sources** | **Intervention and comparator(s)** | **Stated limitations** | **Stated conclusions** |
| --- | --- | --- | --- | --- | --- | --- | --- | --- |
| Bianchi 2019 | Evaluation of a routine point-of-care intervention for early infant diagnosis of HIV: an observational study in eight African countries | Cost analysis | Cameroon, Côte d’Ivoire, Kenya, Lesotho, Mozambique, Rwanda, Swaziland, and Zimbabwe | Not stated | Ministry of Health registers in Elizabeth Glaser Pediatric AIDS Foundation (EGPAF)-supported sites, health facility registers, and other sources. | PoC EID | (1) Observational, non-controlled before-and-after study, that did not control for time and other secular trends. (2) Costs used for both POC and conventional EID did not include human resource, sample transport, or caregiver out-of-pocket costs. | POC EID is both feasible and effective to optimise national EID networks and is an innovative investment that improves service delivery and clinical outcomes, with more results returned to caregivers, earlier ART initiation, and potential reductions in morbidity and mortality for infants with HIV. |
| Collins 2014 | Modeling the Performance and Cost of Early Infant HIV Diagnosis at Birth | Cost-effectiveness (abstract) | South Africa | Not Stated | Not specified in abstract | Birth + 6 week EID vs. Standard 6-week EID | Not specified in abstract. | EID at birth would potentially increase the proportion of HIV-infected children diagnosed, but has lower PPV. If not accompanied by improved retention and referral for ART, it offers limited improvements in proportion starting ART or reducing pre-ART mortality. |
| Collins 2014 | Cost-effectiveness of early infant HIV diagnosis of HIV-exposed infants and immediate antiretroviral therapy in HIV-infected children under 24 months in Thailand | Cost-effectiveness | Thailand | Health system | Thai observational cohort (PHPT paediatric observational cohort study), Thai national EID programme, literature. | EID with immediate ART vs. (1) deferred ART. (2) Clinical diagnosis and deferred ART | (1) Survival estimates of children on ART extrapolated from an observational study with five years of follow-up. (2) Markov model of children on ART assumed non reversibility of health states. (3) Findings not generalizable to other settings. (4) Assumed 100% sensitivity and specificity of DNA PCR testing from 2 months of life. | Early infant HIV diagnosis combined with immediate ART of children under 24 months was cost effective in the Thai setting as compared to late diagnosis and deferred treatment. |
| De Broucker 2021 | The cost-effectiveness of scaling-up rapid point-of-care testing for early infant diagnosis of HIV in southern Zambia | Cost-effectiveness | Zambia | Not stated | (1) Epidemiological model parameters: Literature and NSEBA study. (2) Cost data: NSEBA study and CHAI | PoC EID vs. conventional laboratory EID | (1) Variations in parameters may exist between settings. (2) Did not consider longer term treatment outcomes and costs. | PoC testing has the potential to significantly improve linkage to care and early ART initiation for HIV-infected infants. Integrating use of PoC platforms across disease programs will increase their cost- effectiveness. |
| Dugdale 2019 | Cost-effectiveness of integrating postpartum antiretroviral therapy and infant care into maternal and child health services in South Africa | Cost-effectiveness | South Africa | Health system | Maternal and Child Health–Antiretroviral Therapy (MCH-ART) randomized controlled trial | Integrated vs. separate postpartum maternal and infant care | (1) Uncertainty is inherent in long-term projections. (2) Did not account for subsequent pregnancies that may attenuate budget impact or prompt re-engagement in care. (3) Simulated only breastfeeding women who were not already taking ART at the beginning of pregnancy. | Integrating postpartum maternal and pediatric care improves clinical outcomes at one year, is likely to lead to improved life expectancy among mothers and HIV-infected infants, and is cost-effective in South Africa. This approach to integrated postpartum care should be more widely implemented to improve maternal and child health. |
| Dunning 2017 | The value of confirmatory testing in early infant HIV diagnosis programmes in South Africa: A cost-effectiveness analysis | Cost-effectiveness | South Africa | Health system | Published trials and cohort studies in sub-Saharan Africa, CHAI price lists | Confirmatory EID testing vs. No confirmatory EID | (1) Uncertainty in long-term projections. (2) Model structure does not permit formal probabilistic sensitivity analyses. (3) Did not include negative clinical impacts for HIV-uninfected infants on ART. | Confirmatory testing in EID programmes substantially reduces the proportion of infants incorrectly diagnosed and initiated on ART. While projected cost differences are small, confirmatory testing is cost- saving under a wide range of scenarios in South Africa. |
| Dunning 2021 | Optimizing infant HIV diagnosis with additional screening at immunization clinics in three sub-Saharan African settings: a cost-effectiveness analysis | Cost-effectiveness | Cote d'Ivoire, South Africa, and Zimbabwe | Health system | Clinical data from published trials and cohort studies in sub-Saharan Africa, UNAIDS, UNICEF country-specific data. | Screening for HIV exposure at first infant immunization visit vs. Standard 6-week EID | (1) Long-term model-based projections for children are uncertain. (2) Did not evaluate clinical outcomes, costs or potential reduced MTCT in subsequent pregnancies among mothers. (3) Costs do not account for societal costs incurred or offset as a result of a screen-and-test strategy. (4) No simulation of alternative approaches to screen- and-test. | Screening for infant HIV exposure at the first immunization visit, followed by NAT for exposed infants, would decrease MTCT among infants whose mothers are undiagnosed or not virologically suppressed on ART, improve LE among infants with HIV, and may be cost-effective in South Africa and Zimbabwe. In a low maternal HIV prevalence setting like Cote d’Ivoire, screen-and-test is less likely to be cost-effective relative to existing health interventions. |
| Finocchario-Kessler 2014 | If you text them, they will come: using the HIV infant tracking system to improve early infant diagnosis quality and retention in Kenya | Cost analysis | Kenya | Not stated | Study health facilities | HITSystem patient tracking | (1) Inability to demonstrate causality. (2) Potential for covariate imbalance between populations in the historical control group and unknown or unmeasured covariates that may have influenced the study outcomes (i.e. improvement in service delivery over time). (3) Restricted generalizability of pilot findings to urban and peri-urban government hospitals. (4) Limited and inconsistent documentation of EID outcomes in the control period. | The HITSystem maximizes the use of easily accessible technology to improve the quality and efficiency of EID services in resource-limited settings. |
| Finocchario-Kessler 2015 | Lessons learned from implementing the HIV infant tracking system (HITSystem): A web-based intervention to improve early infant diagnosis in Kenya | Cost analysis | Kenya | Not stated | Study health facilities and designated regional central laboratories | HITSystem | (1) Efficacy data from most sites limited by the lack of matched control data. (2) Current version of the HITSystem does not address uptake of EID services. | HITSystem outcomes in Kenya consistently exceed EID outcomes from national reports or other research studies assessing current EID practices and outcomes. |
| Frank 2019 | Clinical Impact and Cost-Effectiveness of Incorporating Point-of-Care (POC) Assays into Early Infant HIV Diagnosis (EID) Programs at 6 Weeks of Age in Zimbabwe: A Modelling Study | Cost-effectiveness | Zimbabwe | Health system | Clinical trials and cohort studies in Africa, International Epidemiologic Database to Evaluate AIDS (IeDEA), Cape Town AIDS Cohort, Global Fund, WHO systematic reviews. | PoC EID vs. conventional laboratory EID | (1) Base case analysis assumed 100% EID uptake for POC and conventional EID, overestimating clinical benefit of both modelled strategies. (2) Costing inputs for conventional and POC EID drawn from the Global Fund’s TCO do not include health worker costs and infrastructure upgrades. | Incorporating POC assays into EID programs at 6 weeks of age in Zimbabwe markedly improved survival and life expectancy for HIV-exposed infants, and was cost-effective compared to conventional EID. |
| Francke 2016 | Clinical Impact and Cost-effectiveness of Diagnosing HIV Infection During Early Infancy in South Africa: Test Timing and Frequency | Cost-effectiveness | South Africa | Health system | African trials and cohort studies, WHO systematic reviews, South African health system, published data, ART cost price lists, and expert opinion. | Birth + 6 week EID vs. (1) 6 week EID. (2) No EID | (1) Long-term model-based projections uncertain. (2) Not able to fully assess economic trade-offs of scaling up existing 6 weeks alone strategy, compared with adding testing at birth. (3) Maternal HIV status may not be known or adequately documented, or maternal infection may occur after antenatal HIV testing. | A 6 weeks alone strategy markedly improve infant outcomes and are of good value in South Africa as compared to a no EID strategy. Testing twice, at birth and 6 weeks of age, will further improve outcomes and be cost-effective when uptake is high. |
| Jani 2010 | Innovative expedited results system (ERS) greatly improves quality of Mozambique early infant diagnosis program | Cost analysis (abstract) | Mozambique | Not stated | Study health facilities | ERS and simple, inexpensive printers for result transmission | Not stated in abstract. | Decrease in the average return time of results to patients at insignficant cost, even at high volume. Expected that the effect of the ERS will be even more dramatic for less-resourced and more distant health centers. |
| Khamadi 2008 | Rapid Identification of Infants for Antiretroviral Therapy in a Resource Poor Setting: The Kenya Experience | Cost analysis | Kenya | Not stated | Study health facilities | Standard laboratory EID | Not listed. | EID by DNA PCR is feasible from a technical and infrastructural standpoint, but costs per test are prohibitive and could easily be unaffordable for the majority of the population without financial support. |
| Kiyaga 2012 | Mobile phone follow-up is affordable and effective in reducing loss to follow-up among HIV-exposed infants in Uganda | Cost analysis (abstract) | Uganda | Health system | EID registry data from health facilities | Mobile phone vs. home visits for follow-up EID services | Not stated in abstract. | Phone follow-up is effective at in bringing HIV-exposed infants back into care in Uganda at lower costs than home visits, enabling follow-up of larger patient volumes and more frequent follow-up. |
| Kiyaga 2013 | Uganda’s New National Laboratory Sample Transport System: A Successful Model for Improving Access to Diagnostic Services for Early Infant HIV Diagnosis and Other Programs | Cost analysis | Uganda | Not stated | Study health facilities | Novel specimen transport network system | The HUB network system provided increased access to EID services ranging from 36% to 51%, drastically reduced transportation costs by 62%, reduced turn-around times by 46.9% and by a further 46.2% through introduction of SMS printers. | The HUB model provides a functional, reliable and efficient national referral network to increase access to critical diagnostic and treatment services, improve quality of laboratory and diagnostic services, reduce turn-around times and reduce long-term costs. |
| Kiyaga 2015 | Consolidating HIV testing in a public health laboratory for efficient and sustainable early infant diagnosis (EID) in Uganda | Cost analysis | Uganda | Not stated | EID database, patient chart reviews, program work plans, and bills from EID testing labs before lab consolidation. | (1) Single, national vs. (2) Four-laboratory system vs. (3) Eight-laboratory system for EID | Not listed. | Laboratory consolidation has increased the sustainability of HIV testing for infants. |
| Martin 2017 | Early infant diagnosis of HIV-1 infection in Luanda, Angola, using a new DNA PCR assay and dried blood spots | Cost analysis | Angola | Not stated | Angolan Pediatric HIV Cohort (APEHC), Hospital Egas Moniz, Angolan National Institute of Public Health | Proviral DNA PCR-based assay (in-house) for EID from DBS | No head-to-head comparison of assay with a commercial test. | High analytical and clinical sensitivity of the EID assay enabling accurate, early and low cost diagnosis of HIV-1 infection in exposed infants in Angola. |
| McCann 2020 | Strengthening Existing Laboratory-Based Systems vs. Investing in Point-of-Care Assays for Early Infant Diagnosis of HIV: A Model-Based Cost-Effectiveness Analysis | Cost-effectiveness | Zimbabwe | Health system | UNAIDS, International Epidemiologic Database to Evaluate AIDS (IeDEA) East African data, Cape Town AIDS Cohort, P1060 trial, literature. | PoC EID vs. strengthened laboratory EID vs. conventional laboratory EID | (1) Long- term model-based projections are uncertain. (2) Assumed 100% EID uptake among HIV-exposed infants for all strategies. (3) Clinical or economic impact of using POC EID at birth was not evaluated. | The analysis demonstrated that incorporating POC assays into EID programs at 6 weeks of age in Zimbabwe would reduce early mortality, increase LE, and be a more efficient use of resources than strengthening existing laboratory-based EID programs. |
| Menzies 2009 | Cost-Effectiveness of Routine Rapid Human Immunodeficiency Virus Antibody Testing Before DNA-PCR Testing for Early Diagnosis of Infants in Resource-Limited Settings | Cost-effectiveness | Uganda | Program prospective | Pilot infant HIV testing programs at Tororo District Hospital and Mulago National Referral Hospital. | RHT screening before conventional laboratory EID vs. No RHT screening | (1) Lack of explicit ICER threshold. (2) Lack of data regarding health related quality of life. (3) Lack of a clear time horizon. | Screening infants with RHT before DNA-PCR is cost- effective in infants 3 months or older. Incorporating RHT into early infant testing programs could improve cost-effectiveness and reduce program costs. |
| Mukherjee 2020 | Estimating the Cost of Point-of-Care Early Infant Diagnosis in a Program Setting: A Case Study Using Abbott m-PIMA and Cepheid GeneXpert IV in Zimbabwe | Cost analysis | Zimbabwe | Health system | Project expenditures, time-use observations of health workers, and government salary scales | PoC EID | Sample transport costs and cost of adding more machines where volume exceeds the capacity of a single platform excluded. | POC EID cost per test was estimated at $44.55. The results would support recent analysis indicating that POC EID is of excellent value relative to central laboratory-based EID. |
| Ndulue 2015 | Improving dried blood spot transport logistics for early infant diagnosis in Nigeria: the SPEEiD model | Cost analysis (abstract) | Kwara state, North Central Nigeria | Not stated | Not listed | Improved transport logistics for EID (SPEEiD) | Not specified in abstract. | The MSH SPEEiD model remains a cost effective, sustainable and time-sensitive sample transfer model. |
| Salvatore 2021 | Modeling the cost-effectiveness of point-of-care platforms for infant diagnosis of HIV in sub-Saharan African countries | Cost-effectiveness | sub-Saharan African countries | EID program | Seth McGovern and CHAI | PoC EID vs. conventional laboratory EID | (1) Did not consider treatment outcomes or costs, which may underestimate long-term costs and benefits of PoC testing. (2) Variations in parameters may exist between settings. (3) Did not consider alternative intervention scenarios in which the performance of centralized labs was improved. | PoC testing programs can expect significant clinical, epidemiological, and social benefits, supporting implementation of PoC platforms for EID across a range of settings. PoC implementation will likely require significant investment but allowing integrated use of platforms can decrease the costs for EID programs. |
| Sutcliffe 2021 | The NSEBA Demonstration Project: implementation of a point-of-care platform for early infant diagnosis of HIV in rural Zambia | Cost analysis | Zambia | Not stated | Study health facilities | PoC EID | (1) Relatively small number of tests and number of HIV- infected children. (2) Limited number of facilities in one rural area of Zambia. (3) Study carried out with dedicated staff and funds so may overestimate the impact of PoC testing. | Implementing PoC testing in rural health centres in Zambia required significant initial and ongoing investment in infrastructure, training and supervision. |
| Sutcliffe 2021 | Point-of-care p24 antigen detection for early infant diagnosis of HIV infection: cross-sectional and longitudinal studies in Zambia | Cost analysis | Southern Province, Zambia | Not stated | Study health facilities | Novel point-of-care p24 antigen detection test (LYNX) | Not listed. | LYNX had low sensitivity at birth but moderate sensitivity after 4 weeks of age. Given its high specificity, affordability, and robustness, the LYNX test could play an important role in EID to increase linkage to care for HIV-infected infants ≥4 weeks of age at smaller clinics and in remote areas where implementing NAT is not feasible. |
| Tchuenche 2018 | Estimating the cost of diagnosing HIV at birth in Lesotho | Cost analysis | Lesotho | Not stated | Study health facilities | Birth EID | (1) Personnel salaries, utility and construction costs, demographics of participants, cost of quality assurance/improvement, cost savings per infection averted data not available for all facilities. (2) Relied on recall to assess how staff spent their time on VEID activities. | Cost-per-identified child is low and reduction in early mortality high in settings with high in utero infeciton rates. Universal birth testing recommended for settings where PMTCT programs are not well-established and HIV in utero infection rates are high. Targeted testing program recommended for other settings. |
| Terris-Prestholt 2020 | The potential for quality assurance systems to save costs and lives: the case of early infant diagnosis of HIV | Cost-effectiveness | Kenya, Senegal, South Africa, Uganda, Zimbabwe | Health system | Costs modelled from existing data sources in each respective country. | Quality assurance system vs. No quality assurance system | (1) Scarcity of published data across settings for POCT for EID on the observed rates of misdiagnoses and the share of false positives and false negatives. (2) Only accounted for the excess treatment cost associated with a false-positive result. (3) Only explored LtFU in sensitivity analysis for reducing treatment costs. (4) Assumed all HIV transmission occured prior to testing. | If the quality assurance system reduces misdiagnosis from as low as 5%, it has potential to save lives and costs in most settings. Ongoing support for PoC EID is critical to ensure it fulfils its potential for alleviating testing bottlenecks. |
| Touré 2013 | Public sector services for the prevention of mother-to-child transmission of HIV infection: a micro-costing survey in Namibia and Rwanda | Cost analysis | Namibia and Rwanda | Health system | Study health facilities | Package of PMTCT interventions | (1) Activities that support PMTCT but fall outside national PMTCT programmes were ignored. (2) Operating costs only. (3) To estimate national costs, unit costs were multiplied by the reported numbers of individuals using PMTCT services in each country. (4) Assumed individuals reached by the national PMTCT programmes attended only facilities that provided those services. | Costs of PMTCT varied widely across countries, but per-capita costs were relatively low, and therefore scaling up PMTCT services in Namibia and Rwanda should be possible. |
| Vyas 2020 | Cost variations in prevention of mother-to-child HIV transmission services integrated within maternal and child health services in rural Tanzania | Cost analysis | Tanzania | Health system | Health Management Information Systems, service use data from facility records, facility survey, Global Fund, National AIDS Control Programme | Neonatal HIV care including Nevirapine prophylaxis and standard 6-week EID | (1) Only captures provider costs for core PMTCT services. (2) Data on staff time from interviews rather than direct observation. | PMTCT costs vary across type of health facility and scale of operation. With high birth rate and declining HIV positivity rate, average cost per case diagnosed within PMTCT services will likely increase. Careful planning in allocation of resources, especially personnel, needed to ensure value for money when meeting increased demand. |
| Zegeye 2019 | Assessing the cost of providing a prevention of mother-to-child transmission of HIV/AIDS service in Ethiopia: Urban-rural health facilities setting | Cost analysis | Ethiopia | Health system | Study health facilities | PMTCT services | (1) Snapshot of costing results across the heterogeneous HIV prevalence and urban-rural settings. (2) Facilities used to estimated national PMTCT service cost not randomly selected.. | Resources used for PMTCT service packages varied across health facilities and HIV prevalence contexts. Context-specific costing vital to provide locally sensitive evidence for health service management and priority setting. |
